# Analytical bias of automated immunoassays for six serum steroid hormones assessed by LC-MS/MS

**DOI:** 10.11613/BM.2020.030701

**Published:** 2020-08-05

**Authors:** Željko Debeljak, Ivana Marković, Jasna Pavela, Iva Lukić, Dario Mandić, Sanja Mandić, Vesna Horvat, Vatroslav Šerić

**Affiliations:** 1 Clinical Institute of Laboratory Diagnostics, Osijek University Hospital, Osijek, Croatia; 2 Faculty of Medicine, JJ Strossmayer University of Osijek, Osijek, Croatia

**Keywords:** steroid hormone, immunoassay, bias, LC-MS/MS, method comparison

## Abstract

**Introduction:**

There is a growing amount of evidence showing the significant analytical bias of steroid hormone immunoassays, but large number of available immunoassays makes conduction of a single comprehensive study of this issue hardly feasible. Aim of this study was to assess the analytical bias of six heterogeneous immunoassays for serum aldosterone, cortisol, dehydroepiandrosterone sulphate (DHEAS), testosterone, 17-hydroxyprogesterone (OHP) and progesterone using the liquid chromatography coupled to the tandem mass spectrometry (LC-MS/MS).

**Materials and methods:**

This method comparison study included 49 serum samples. Testosterone, DHEAS, progesterone and cortisol immunoassays were performed on the Abbott Architect i2000SR or Alinity i analysers (Abbott Diagnostics, Chicago, USA). DiaSorin’s Liaison (DiaSorin, Saluggia, Italy) and DIAsource’s ETI-Max 3000 analysers (DIAsource ImmunoAssays, Louvain-La-Neuve, Belgium) were chosen for aldosterone and OHP immunoassay testing, respectively. All immunoassays were evaluated against the LC-MS/MS assay relying on the commercial kit (Chromsystems, Gräfelfing, Germany) and LCMS-8050 analyser (Shimadzu, Kyoto, Japan). Analytical biases were calculated and method comparison was conducted using weighted Deming regression analysis.

**Results:**

Depending on the analyte and specific immunoassay, mean relative biases ranged from -31 to + 137%. Except for the cortisol, immunoassays were positively biased. For none of the selected steroids slope and intercept 95% confidence intervals simultaneously contained 0 and 1, respectively.

**Conclusions:**

Evaluated immunoassays failed to satisfy requirements for methods’ comparability and produced significant analytical biases in respect to the LC-MS/MS assay, especially at low concentrations.

## Introduction

Immunoassays are widely used for quantification of different serum steroid hormones. This analytical approach can be easily automated which, in turn, reduces the turn-around-time and staff requirements. As such, serum steroid immunoassays are especially suitable for the diagnostic workup of patients coming to emergency rooms. However, over the last few decades’ awareness about the flaws of immunoassays for quantification of biomolecules is growing (*1*). While the immunoassays for protein quantifications are prone to different types of interferences, immunoassays intended for determinations of small biomolecules like steroids are affected primarily by the lack of selectivity (*2*, *3*). Common interfering molecules in immunoassays are metabolites and drugs (*3*). In the classical congenital adrenal hyperplasia (CAH), falsely elevated cortisol may be due to the cross-reactivity with 21-deoxycortisol, while in the pregnancy pregnenolone sulphate interferes with the dehydroepiandrosterone sulphate (DHEAS) measurement. Falsely elevated cortisol may be encountered during the hypoadrenalism treatment with prednisolone or 6-metylprednisolone. Also, estradiol immunoassays may show striking method-specific bias in lower serum concentration range, for example in aromatase-inhibitor treated women (*2*).

Antibodies mostly do not have strong affinity for small molecules such as steroid hormones and the differences between the small structurally related molecules having different biological roles cannot be safely discerned using antibodies (*4*, *5*). Numerous endogenous and exogenous compounds sharing the same sterane scaffold may exist in serum or other biological fluids and some of them may even have the same empirical formula. Similar functional groups and short side chains attached to the sterane positions that are in close proximity can be hardly recognized by the macromolecular structures like antibodies (*5*).

This lack of selectivity reflects itself on quantification accuracy, which causes the analytical bias. First reports on steroid immunoassays bias date back to late 1990’s (*1*). In these cases, the bias has been assessed using mostly gas chromatography – mass spectrometry (GC-MS). Problems with the analytical bias of serum immunoassays for steroid quantification still persist: there is a long-lasting demand for more accurate methods and techniques that are less complex in terms of manual sample preparation than GC-MS (*1*-*5*). Techniques like the liquid chromatography – tandem mass spectrometry (LC-MS/MS) with electrospray ionization source are better suited for the analysis of steroid hormones in body fluids (*6*). Multiple reaction monitoring (MRM) mode of tandem mass spectrometry (MS) combined with the chromatographic separation of different serum components significantly improves the analytical selectivity required for differentiation between closely structurally related steroids. Moreover, visual presentation of results in the form of chromatogram allows the analyst to detect possible interferences. Detection and even elimination of possible problem associated with interferences can be achieved using different MRM channels for the same compound (*5*, *6*). Multiple reaction monitoring also enables application of the isotopically labelled internal standards (ISTD), which improves the accuracy even more. All these properties of the LC-MS/MS technology make it reliable reference against which the analytical bias of less selective analytical techniques may be assessed.

Due to the vast number of steroids and ever-emerging immunoassays, most of the assays available on the market have not been sufficiently evaluated utilizing more selective techniques like isotope dilution mass spectrometry and/or LC-MS/MS. This is the case even for the frequently utilized steroid assays (*7*, *8*). Moreover, the majority of existing studies of analytical bias were focused on a single steroid hormone and a single immunoassay: there is a need for more elaborate approach to the problem covering different immunoassays and different steroid hormones which will enable some general conclusions to be drawn (*6*-*8*). The aim of this study is to analyse the analytical bias of heterogeneous immunoassays for six serum steroid hormones against the validated and commercially available LC-MS/MS assay. The LC-MS/MS assay enables multiplex quantification of serum steroids, among which aldosterone, cortisol, DHEAS, testosterone, 17-hydroxyprogesterone (OHP) and progesterone were selected for the analytical bias evaluation. Automated heterogeneous immunoassays, that is sandwich ELISA assay, chemiluminescent microparticle immunoassay (CMIA) and chemiluminescent immunoassay (CLIA) were evaluated in this study. This particular immunoassay and analyser platform selection reflects the need for accomplishment of maximum possible diversity of evaluated immunoassays which is the prerequisite for reaching general conclusions. Among steroid hormones frequently measured in both clinical and research settings, only the estradiol was omitted due to reports stating that, under certain circumstances, even the LC-MS/MS assay for estradiol quantification may be prone to interferences (*2*).

## Materials and methods

### Subjects

This method comparison study was conducted at the Clinical Institute of Laboratory Diagnostics, Osijek University Hospital, Croatia from September to December 2018 in accordance with the Declaration of Helsinki. The study was approved by the Hospital’s Ethical Committee. Patients being routinely evaluated for the serum steroid hormone status were enrolled in the study (N = 65): this was the sole inclusion criterion. The preliminary diagnoses of enrolled patients consisting of 37 females aged 32 (19–45) and 12 males aged 37 (27–62) were postulated by a physician as: female and male infertility, testicular and ovarian disorder, polycystic ovary syndrome, amenorrhea, acne and benign adrenal neoplasm. One sample *per* patient has been analysed by all immunoassays and by the LC-MS/MS assay. Due to the following exclusion criteria: insufficient sample volume, haemolysis, lipemia or icterus 15 samples were excluded. A total of 49 samples were used for the evaluation, which is in accordance with CLSI EP09-A3 guidelines: Measurement Procedure Comparison and Bias Estimation Using Patient Samples (3^rd^ edition).

After an overnight fasting, one blood sample *per* each enrolled patient was drawn from the antecubital vein. The sampling was done between 7 and 9 hours a.m. in 7 mL tubes containing no anticoagulant or gel separator (Becton Dickinson, Franklin Lakes, USA), where upon they were left in the upright position for 30 minutes before centrifugation at 2000xg for 10 minutes at room temperature using 3-16PK centrifuge (Sigma Laborzentrifugen, Osterode am Harz, Germany). Each serum was separated from the clot, divided into 600 µL aliquots and stored at - 18°C up to one month until the analysis.

### Methods

Samples for the multiplex LC-MS/MS analysis of aldosterone, cortisol, testosterone, DHEAS, progesterone and OHP were prepared according to the kit manufacturer’s instructions (Chromsystems, Gräfelfing, Germany). Prepared samples were analysed using Nexera X2 liquid chromatograph coupled with Shimadzu LCMS-8050 tandem mass spectrometer (Shimadzu, Kyoto, Japan) equipped with the electrospray ionization source. According to the LC-MS/MS kit manufacturer’s instructions, analyses were conducted using two different instrumental settings and two different sets of calibrators and controls, which is two different panels. The first panel was intended for the aldosterone and cortisol determinations and the second was intended for the DHEAS, testosterone, progesterone and OHP determinations. Both panels shared the same sample preparation steps and the same instrumentation. All calibrators were traceable to certified reference materials and primary standards. For each analyte the kit manufacturer provided corresponding isotopically labelled ISTD and at least two MRM channels for each analyte and corresponding ISTD. Implementation of the kit on the LC-MS/MS instrument used in this study has been done by the manufacturer’s application specialist: all chromatography settings and all MS/MS settings were implemented according to the recommendations specific for the LC-MS/MS instrument used in this study. Mass transitions tuning has been done using tuning mixes provided by the manufacturer. Method validation data obtained on two different LC-MS/MS instruments provided by the manufacturer are summarized in Table 1.

**Table 1:** Summary of the LC-MS/MS assay validation data provided by kit manufacturer

**Analyte, unit** **(polarity: MRM1; MRM2)**	**Reagent/** **Manufacturer**	**Assay range**	**Limit of** **quantification**	**Inter-assay** **precision, CV%**	**Recovery, %**
Aldosterone, nmol/L (-:359®331;359®189)	MassChrom Steroids in Serum and Plasma/ Chromsystems	0.039–16.62	0.039	5.9%	95%
Cortisol, µmol/L (+:363®97;363®121)		0.004–1.44	0.004	5.0%	93%
DHEAS, µmol/L (-:369®99;369®82)		0.066–48.78	0.066	6.9%	90%
Testosterone, nmol/L (+:289®97;289®109)		0.02–83.28	0.02	8.9%	95%
Progesterone, nmol/L (+:315®97;315®109)		0.095–79.5	0.095	10.2%	82%
OHP, nmol/L (+:331®109;331®97)		0.12–90.9	0.12	9.5%	92%
Both MRM channels were validated using two different LC-MS/MS instruments. LC-MS/MS - liquid chromatography coupled to the tandem mass spectrometry. MRM - multiple reaction monitoring. DHEAS - dehydroepiandrosterone sulfate. OHP - 17-hydroxyprogesterone.					

Testosterone, DHEAS and cortisol immunoassays were performed using commercially available Abbott CMIA kits implemented on the Abbott Architect i2000SR, while progesterone immunoassay was performed on Abbott Architect i2000SR and Abbott Alinity i automatic analysers (Abbott Diagnostics, Chicago, USA). Aldosterone concentration was measured using CLIA method implemented on DiaSorin Liaison automatic analyser (DiaSorin, Saluggia, Italy), while OHP concentration was determined by DIAsource sandwich ELISA method (DIAsource ImmunoAssays, Louvain-La-Neuve, Belgium) implemented on the ETI-Max 3000 (DiaSorin, Saluggia, Italy). Manufacturers provided calibrators, internal control materials and method validation reports (Table 2) for all commercially available kits. All immunoassay calibrators were traceable to primary standards and certified reference materials.

**Table 2:** Summary of the immunoassay validation data provided by kit manufacturers

**Analyte, unit**	**Reagent/** **Manufacturer**	**Assay range**	**Limit of** **quantification**	**Total precision, CV %**	**Recovery, %**
Aldosterone, nmol/L	Liaison Aldosterone/ DiaSorin	0.027–2.77	0.053	9.5%	91%
Cortisol, µmol/L	Alinity i Cortisol/ Abbott	0.028–1.650	0.028	3.7%	n.a.
DHEAS, µmol/L	Architect DHEA-S/ Abbott	0.08–40.71	n.a.	7.41%	102%
Testosterone, nmol/L	Architect 2^nd^ gen Testosterone/Abbott	0.13–64.57	0.15	4.6%	n.a.
Progesterone, nmol/L	Alinity i Progesterone/ Abbott	1.6–127.2	1.6	5.8%	n.a.
	Architect Progesterone/ Abbott	n.a.–127.2	n.a.	6.2%	96%
OHP, nmol/L	17-OH-progesterone/ DIAsource	0.10–60.52	n.a.	n.a.	105%
DHEAS - dehydroepiandrosterone sulfate. OHP - 17-hydroxyprogesterone. n.a. = not available					

For all analytes and assays, internal quality control provided by each manufacturer has been performed as a part of every analytical sequence, while the external quality control evaluation of all analytes has been carried out on a monthly basis using Randox Riqas Monthly Immunoassay scheme (Randox, Crumlin, UK). Internal quality control material for the LC-MS/MS assay included 5 concentration levels for each analyte and, acceptance limits set to 80–120% recovery by the kit manufacturer, were met in each analytical sequence for each MRM channel. Besides the internal quality control, the system suitability parameters evaluated in each analytical sequence included also calibration regression coefficient (r) and ISTD coefficient of variation (CV%). For each analyte the calibration r obtained on 7 concentration levels was ≥ 0.99 and recoveries calculated for each calibration level were 80–120%. Internal standard CV% calculated for all samples in the analytical sequence were ≤ 35% for each analyte. Finally, for the selected MRM no peak splitting or other sources of peak asymmetry which may indicate existence of interfering substances were detected for any analyte in any of the recorded chromatograms.

### Statistical analysis

Two approaches to immunoassay performance evaluation were applied (*9*, *10*). The first relies on the absolute bias (AB) and relative bias (RB) which was calculated according to Equation (Eq.) 1:
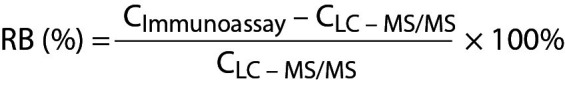
where *C* stands for the serum steroid concentration measured either by a selected immunoassay or by the LC-MS/MS assay.

Acceptance criterion for the mean relative bias (*RB*) was set to ± 20%. This choice stems from the acceptance criteria for analytical accuracy that is recovery of LC-MS/MS assay used which is 80 - 120%. Published desirable bias intervals for aldosterone, testosterone, OHP, cortisol and DHEAS are all narrower than 80–120%, making the chosen interval quite inclusive (*11*). Besides the *RB*, mean absolute bias (*AB*) has been calculated and graphically presented in the form of a Bland-Altman plot.

The second approach to immunoassay evaluation relies on the weighted Deming regression accompanied by the jack-knife method for 95% confidence interval (CI) estimation (*12*). In this case, two acceptance criteria had to be met to reach a conclusion about the analytical method comparability. These criteria involved 95%CI for slope and intercept which should contain values 1 and 0, respectively. Otherwise, analysed methods should be regarded as non-comparable. As a part of the regression analysis, proportional bias (PB) (%) together with the corresponding 95%CI has been estimated using Equation 2 (*13*).


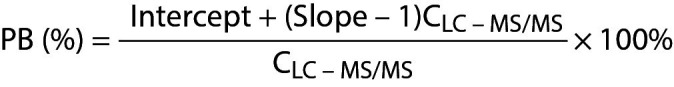


Instead of a simple Deming regression, the weighted version has been selected due to its robustness towards heteroscedasticity (*12*, *14*). For evaluating weighted Deming regression suitability for the analytical method comparison, the Pearson correlation coefficient together with the test of normality of standardized residuals and CUSUM test of linearity were conducted, both relying on the Anderson-Darling statistics. All calculations and graphical representations of the results were performed using R package for statistical computing v. 3.1.2. (R Foundation for Statistical Computing, Vienna, Austria) together with the mcr contributed package (*15*). If not stated otherwise, the default values were used for computations. P < 0.05 was considered significant.

## Results

**Figure 1:**
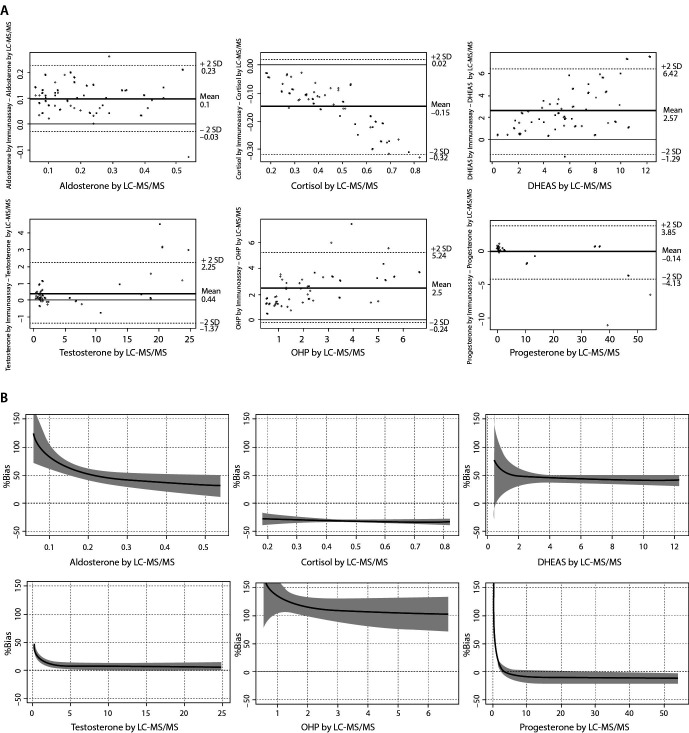
Graphical representation of immunoassay’s analytical bias estimated using the liquid chromatography coupled to the tandem mass spectrometry assay (N = 49). A: Bland-Altman plots showing AB together with the corresponding trends and dispersions. B: Estimated PB and corresponding 95% Confidence interval (95%CI). All concentrations are given in nmol/L, except for the cortisol and DHEAS which are given in µmol/L. DHEAS - dehydroepiandrosterone sulphate. OHP - 17-hydroxyprogesterone.

Mean relative bias and corresponding coefficients of variations (CV%) were calculated for all analytes and averaged over all samples (Table 3). Since the *RB* and its dispersion may vary along the concentration axis, this assumption was tested using the Bland-Altman plot (Figure 1A) and plot of the PB (Figure 1B). The Bland-Altman plot for cortisol shows a descending trend in AB and negative PB, even at the lowest concentrations. In the OHP and DHEAS cases, the AB and its dispersion rise along the axis, while the PB decreases and then stabilizes along the concentration axis indicating the important contribution of the PB to the total bias at low concentrations. In the progesterone case, both AB and PB change the sign along the concentration axis. In all other cases, the biases kept the positive sign. Further along, aldosterone immunoassay also yielded a large positive PB with decreasing tendency which is not stabilizing along the concentration axis.

**Table 3:** Relative bias of analysed serum steroid immunoassays calculated against the LC-MS/MS assay and quantitative results of corresponding weighted Deming regression

**Analyte, unit**	RB% (CV%)*	**Linearity (P)**	**Normality of residuals (P)**
Aldosterone, nmol/L	71.22 (80.71)	0.211	0.890
Cortisol, µmol/L	- 31.14 (33.39)	0.237	0.110
DHEAS, µmol/L	47.88 (58.82)	0.630	0.087
Testosterone, nmol/L	20.81 (139.61)	0.462	0.009
Progesterone, nmol/L	114.76 (139.83)	0.209	0.645
OHP, nmol/L	136.96 (55.71)	0.305	0.682
*Relative bias (RB) was averaged over the complete concentration interval and represented together with the corresponding CV%. P < 0.05 was considered statistically significant. DHEAS - dehydroepiandrosterone sulfate. OHP - 17-hydroxyprogesterone.			

**Figure 2:**
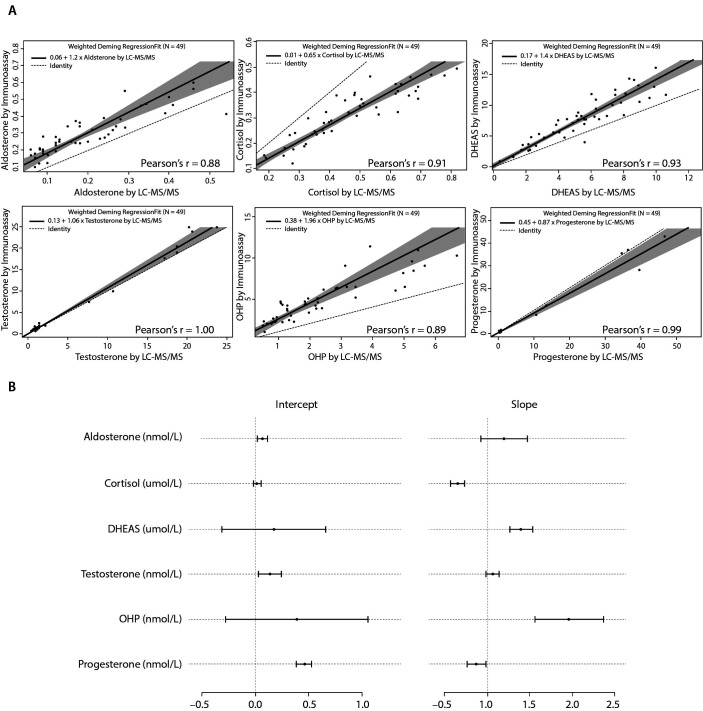
Comparison of serum steroid immunoassays with liquid chromatography coupled to the tandem mass spectrometry assay using weighted Deming regression accompanied by jack-knife based 95% confidence interval (95%CI) estimation (N = 49). A: Regression lines with corresponding 95%CI bands (gray area). Dotted lines represent identity lines. B: Graphical presentation of slopes’ and intercepts’ CI’s. DHEAS - dehydroepiandrosterone sulfate. OHP - 17-hydroxyprogesterone.

Reliability of PB estimated using regression depends on the same assumptions as the regression analysis shown in Figure 2 and Table 3. Linear relationship between all immunoassay and LC-MS/MS measurements was statistically confirmed. Standardized residual’s distribution wasn’t normal only in the case of testosterone immunoassay, what may be attributed to the application of weighted Deming regression. This is especially important for the progesterone which has been determined using two different instruments interchangeably: in such settings, one may expect significant breaching of assumptions about the linearity and the normality distribution of standardized residuals. Furthermore, Figure 2A shows a significant deviation of the regression lines from identity line which indicates the non-comparability of analysed immunoassays and the LC-MS/MS assay. This is confirmed by results shown in the Figure 2B and Table 3: none of the analysed immunoassays produced results comparable to the results produced by the LC-MS/MS assay.

## Discussion

As shown in Tables 1 and 2, all immunoassays as well as the LC-MS/MS assay were validated by manufacturers and they, reportedly, met the validation acceptance criteria. However, our results show large discrepancies between all immunoassays and the LC-MS/MS assay. The discrepancies in terms of analytical bias were detected for six different steroid hormone concentrations assessed by different technologies: this provides the basis for some generalizations. Changes in steroid hormone concentration of less than 20%, as expected during the hormone suppression therapy, exceed the steroid hormone biological variation and may affect the patient’s treatment (*11*). Although immunoassay recovery data, reported by the manufacturer, were within ± 10%, our results show biases larger than ± 20% for all analytes. Analytical bias larger than the ± 20% stems from the lack of analytical method selectivity. For the most of immunoassays, the PB is high in the low concentration range and it decreases as the actual steroid concentration increases. This is consistent with the expected impact of the interfering substances on the steroid hormone determinations: chances are low that the interferents would make a significant contribution to a result produced by the immunoassay if the actual steroid hormone concentration is high. In this case, the PB is expected to be insignificant. But the interferent relative contribution to the result rises as the true steroid concentration decreases.

Considering the reported lack of selectivity, one might expect that immunoassays’ AB and *RB *would be positive in all cases. However, the AB and *RB* of cortisol CMIA were negative. The AB shows a uniform negative trend along the concentration axis which is consistent with the almost straight line accompanied by the narrow 95%CI in the PB plot. This pattern of the PB suggests that cortisol CMIA is less prone to interferences which is also confirmed by the CV% of the *RB*. Still, cortisol CMIA performance, either in terms of bias or in terms of regression features, was unsatisfactory. Negative bias may be associated with the materials and calibration curves used for the CMIA calibration.

The only serum steroid immunoassay, besides the cortisol CMIA, characterized by the slope 95%CI entirely located in the negative region, is the progesterone CMIA. The impact of negative PB on *RB* is diminished by the predominance of samples coming from patients having low actual progesterone concentration (< 1.0 nmol/L). Progesterone CMIA, at least at low actual progesterone concentrations, yielded expected positive AB and PB. This resulted in the switch of AB sign near the value of 10 nmol/L of progesterone and also in large CV% of *RB*.

Testosterone and DHEAS CMIAs, here implemented exclusively on Abbott’s Architect i2000SR, do not show negative biases as opposed to the assays implemented on Abbott’s Alinity i. Testosterone CMIA yielded nearly acceptable RB and was close to become comparable with the LC-MS/MS. Departure from normality of standardized regression residuals and the predominance of low testosterone (< 2.0 nmol/L) containing samples, might explain the detected differences between testosterone CMIA and LC-MS/MS assay. Described pattern of testosterone CMIA is observed in many other testosterone immunoassays (*16*). In contrast to the testosterone CMIA, OHP ELISA produced large *RB*. All other parameters of method comparison were also beyond acceptable limits. Though to the lesser extent, the DHEAS CMIA showed similar pattern as OHP ELISA, and is also characterized by the unacceptable *RB*. DHEAS immunoassay was not comparable to the LC-MS/MS assay, which is consistent with results reported by others (*17*). Finally, aldosterone CLIA represents an interesting case: although the 95%CI for slope and intercept suggested that this immunoassay is nearly comparable with the LC-MS/MS, its *RB* was still too high. Moreover, aldosterone CLIA PB curve descends very slowly, which may indicate poor selectivity of the assay not only in the low concentration range. Problems with excessive *RB* are shared by some aldosterone radioimmunoassays (*18*).

Described problems with the analytical bias of serum steroid immunoassays may have serious consequences. In hereditary diseases of steroid metabolism or in states characterized by the stimulated or autonomous steroid production, the upper limit of the reference interval (RI) is of the greatest diagnostic importance. In such settings, one may expect that the RB values given here are acceptable at least near the upper RI limit. Testosterone, progesterone and cortisol CMIA may be classified into this category. Unfortunately, aside from cortisol, highly elevated concentrations of testosterone and progesterone are diagnostically important only in a limited number of conditions and treatments. Cortisol CMIA represents the opposite case compared to testosterone and progesterone immunoassays: in this case falsely decreased concentrations are expected to affect the diagnostic performance. Still, in cortisol CMIA case, the RB is almost constant, which enables reasonable RI adjustments. Even the weak request for acceptable RB near the upper RI limit does not hold in the OHP case. This analyte accumulates in blood of patients suffering from CAH, making the OHP measurement important diagnostic test. Unfortunately, large RB characteristic for the OHP immunoassays can lead to false diagnoses, previously described by others (*19*, *20*). Large RB near the upper RI limit is also a characteristic of evaluated DHEAS CMIA (Figure 1B). It is expected that the positive RB, at least in some patients, affects the diagnostic evaluation of patients suffering from the polycystic ovary syndrome. The situation is more pronounced at lower serum steroid concentrations. Apart from the cortisol CMIA, all immunoassays yielded large positive RB in this range. This property should strongly discourage one to use evaluated immunoassays for establishment of diagnoses associated with gland hypofunction or its therapeutic suppression. There are number of cases described in literature showing the harmful effect of falsely elevated serum steroid concentrations on the patient treatment (*21*-*23*). Study of Mandić *et al.* describes the impact of exemestane therapy on falsely elevated serum estradiol measurement, while Stowasser *et al.* discuss the importance of correct aldosterone measurement in patients with hypertension, primary hyperaldosteronism and Addison disease (*21*, *22*).

Our study has two major limitations: relatively small number of samples and uneven distribution of samples *per* different concentration ranges for some steroids. However, weighted Deming regression suitability analysis has shown acceptable distribution of residuals and linear dependence of data, which means that the study limitations didn’t significantly affect at least the regression based analysis of bias.

In conclusion, evaluated immunoassays failed to satisfy requirements for methods’ comparability and produced significant analytical biases in respect to the LC-MS/MS assay, especially at low concentrations. Considering the vast number of steroid immunoassays of scientific and diagnostic interest, studies of corresponding analytical bias should be continued using large, well defined sets of specific patient groups.
